# Maternal Iodine Status is Associated with Offspring Language Skills in Infancy and Toddlerhood

**DOI:** 10.3390/nu10091270

**Published:** 2018-09-09

**Authors:** Maria Wik Markhus, Lisbeth Dahl, Vibeke Moe, Marianne Hope Abel, Anne Lise Brantsæter, Jannike Øyen, Helle Margrete Meltzer, Kjell Morten Stormark, Ingvild Eide Graff, Lars Smith, Marian Kjellevold

**Affiliations:** 1 Institute of Marine Research, P.O box 1870 Nordnes, 5817 Bergen, Norway; lisbeth.dahl@hi.no (L.D.); jannike.oyen@hi.no (J.Ø.); ingr@norceresearch.no (I.E.G.); marian.kjellevold@hi.no (M.K.); 2 Department of Psychology, University of Oslo, 0316 Oslo, Norway; vibeke.moe@psykologi.uio.no (V.M); lrssmth@gmail.com (L.S.); 3 Division of Infection Control and Environmental Health, Norwegian Institute of Public Health, 0456 Oslo, Norway; mariannehope.abel@fhi.no (M.H.A.); annelise.brantsaeter@fhi.no; (A.L.B); hellemargrete.meltzer@fhi.no (H.M.M.); 4 Department of Nutrition, TINE SA, 0902 Oslo, Norway; 5 Regional Centre for Child and Youth Mental Health and Child Welfare, Uni Research Health, Uni Research, P.O box 7810, 5020 Bergen, Norway. E-mail: kjst@norceresearch.no

**Keywords:** Bayley, cohort, pregnancy, iodine status, iodine supplementation, infants, ICP-MS, mild to moderate iodine deficiency, neurodevelopment, urinary iodine concentration

## Abstract

Inadequate iodine status affects the synthesis of the thyroid hormones and may impair brain development in fetal life. The aim of this study was to explore the association between maternal iodine status in pregnancy measured by urinary iodine concentration (UIC) and child neurodevelopment at age 6, 12 and 18 months in a population-based cohort. In total, 1036 families from nine locations in Norway were enrolled in the little in Norway cohort. The present study includes *n* = 851 mother-child pairs with singleton pregnancies, no use of thyroid medication in pregnancy, no severe genetic disorder, data on exposure (UIC) in pregnancy and developmental outcomes (Bayley Scales of Infant and Toddler Development, third edition). Data collection also included general information from questionnaires. We examined associations between UIC (and use of iodine-containing supplements) and repeated measures of developmental outcomes using multivariable mixed models. The median UIC in pregnancy was 78 µg/L (IQR 46–130), classified as insufficient iodine intake according to the WHO. Eighteen percent reported use of iodine-containing multisupplements. A UIC below ~100 was associated with reduced receptive (*p* = 0.025) and expressive language skills (*p* = 0.002), but not with reduced cognitive or fine- and gross motor skills. Maternal use of iodine-containing supplements was associated with lower gross motor skills (b = −0.18, 95% CI = −0.33, −0.03, *p* = 0.02), but not with the other outcome measures. In conclusion, an insufficient iodine intake in pregnancy, reflected in a UIC below ~100 µg/L, was associated with lower infant language skills up to 18 months. The use of iodine-containing supplements was not associated with beneficial effects.

## 1. Introduction

Iodine deficiency (ID) is considered one of the most common nutritional disorders globally and is the world’s largest single cause of preventable brain impairment [1]. Infants and pregnant women are particularly vulnerable population groups [2], as ID may impair normal growth and development of the child, especially affecting brain development [3].

Iodine is an essential nutrient for the synthesis of the thyroid hormones triiodothyronine (T_3_) and thyroxine (T_4_). Dietary iodine is rapidly absorbed and the thyroid gland is dependent on regular and adequate supply through the diet in order to produce these vital hormones [4]. In the fetal brain, inadequate thyroid hormone levels impair differentiation, maturation, cell migration, and myelination [3]. The iodine turnover during pregnancy is increased because the mother synthesizes approximately 50% more T_4_ to maintain maternal euthyroidism and transfer thyroid hormones to the fetus, and because iodine needs to be transferred to the fetus for fetal thyroid hormone production. In addition, the renal iodine clearance increases during pregnancy [5,6]. Critically low levels of thyroid hormones, associated with severe ID, cause neurological damage to the brain, particularly during the fetal and neonatal period, which can result in delayed motor and mental development [7,8,9,10]. Mild to moderate ID may also result in impaired mental development [11,12,13,14].

The World Health Organization (WHO) estimates that women in 21 European countries have inadequate iodine intake in pregnancy (defined as a median UIC < 150 µg/L), while only women in 10 countries are considered to have sufficient intake. No data are available in the remaining 23 countries [15]. In Norway, the public health focus on iodine has moved from endemic ID in certain inland areas at the beginning of the 20th century to the assumption of iodine sufficiency since 1950 [16]. However, there has never been a systematic monitoring program in Norway to confirm this assumption. The estimated iodine intake in the Norwegian population among adults and children shows that the intake of milk, dairy products and seafood contributes with ~80% of the total iodine intake [17,18]. This indicates that certain groups of the population may be at risk of a low iodine intake, e.g., subjects with allergy to milk or fish, vegetarians who do not consume fish, milk and dairy products (vegans), and others with low consumption of milk and fish. Other vulnerable groups that might have low iodine intake are pregnant and lactating women since there is a higher recommended intake of iodine during this period of life [19,20,21]. Data from the Norwegian Mother and Child Cohort Study (MoBa) (*n* = 61,904) showed inadequate iodine intake in pregnant Norwegian women and the authors suggested that sub-optimal iodine intake is a health concern in Norwegian pregnant women [22]. Several recent publications have documented mild to moderate iodine deficiency in pregnant women in different parts of Norway, with a median UIC of 92 µg/L (*n* = 777) in the Oslo area [23] and a median UIC of 75 µg/L (*n* = 197) in the Tromsø area [24]. Additionally, published results from the Litle in Norway cohort showed a median UIC of 85 µg/L (*n* = 954) in pregnancy [25]. Further studies and a better understanding of the consequences of mild to moderate ID in pregnancy on subsequent neurodevelopment in infancy and toddlerhood are scarce. Therefore, with samples and data from the Little in Norway study (LiN), the primary aim of this study was to explore whether maternal iodine status in pregnancy, measured by a spot UIC, was associated with child neurodevelopment up to age 18 months. A secondary aim was to investigate the potential impact of maternal iodine supplement use in pregnancy on the same outcome measures. 

## 2. Materials and Methods 

### 2.1. Study Design, Participants and Enrolment

**Figure 1:**
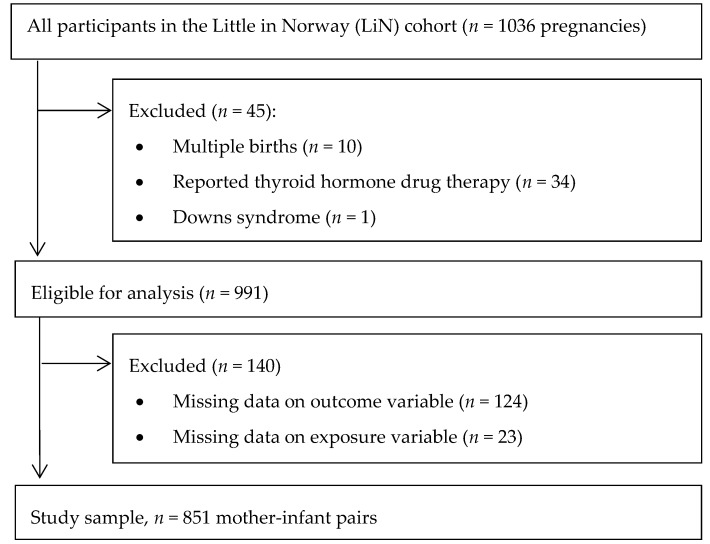
Flow chart showing the study population.

The LiN study is a population-based prospective cohort established to investigate pre- and postnatal risk factors influencing developmental plasticity from pregnancy to age 18 months. Pregnant women were enrolled at nine public health clinics for mothers and children across all four Norwegian health regions. The clinics were chosen after considering demographic characteristics and size of the population to include participants from both cities and rural districts with a wide distribution of socioeconomic conditions. From each site, one public health care nurse was trained as a research assistant. Enrolment started in September 2011 and ended October 2012. Midwives at the public health clinics approached pregnant women 16–26 weeks gestation with an invitation to participate; however, some women were asked as late as weeks 31–34. Data collection up to age 18 months ended in November 2014. Attrition analysis for the mothers in the LiN study is reported by Fredriksen et al. [26]. Inclusion criteria for analysis in the present study were available data on UIC and on child development, singleton pregnancy, no thyroid medication in pregnancy, and no severe genetic disorder (Figure 1).

### 2.2. Data Collection

Participation entailed donating a spot urine sample for analysis of UIC, responding to questionnaires during pregnancy, and meeting with a research assistant after delivery for assessment of child neurodevelopment at 6, 12 and 18 months. The spot urine sample could be collected at home or on site, and be taken at any time. Details regarding time of sampling has been described elsewhere [25]. Data were collected by the research assistants based on interview of the parents, and by testing the child; through self-report questionnaires completed at the public health clinics; and self-report questionnaires completed at home. Details regarding data collection and the questionnaires have been described elsewhere [25,27]

### 2.3. Exposure Variables—Urinary Iodine Concentration and Use of Iodine Containing Supplements

Non-fasting spot-urine samples from the mothers were collected at the first (23.7 ± 5.0 gestational week) and the third (26.4 ± 1.6 gestational week) meeting with the research assistant at the public health clinic. The urine sample collected at the first meeting was preferably used in analysis (*n* = 851; if missing, urine sample collected at the third meeting was used (*n* = 7)). Urinary iodine is regarded as a good population biomarker of iodine intake because more than 92% of ingested iodine is excreted in the urine within 24–48 h [6]. Because it is impractical to collect 24-h samples in field studies, UIC were measured in spot urine collections [2]. Maternal hydration status affects spot-urine concentrations, and since creatinine is excreted in urine at a fairly constant rate, UIC per gram creatinine is often used with the aim to remove variation in UIC due to hydration. UIC in µg/L was used as the primary exposure since creatinine excretion varies in pregnancy with regards to gestational age, maternal age and body composition [6]. Additionally, as alternative exposure measures, we have included, in the online supplementary material, analyses of (1) UIC in µg/g creatinine and (2) residuals of UIC in µg/L after regressing log UIC on log urinary creatinine. 

Questions on supplement use were embedded in a short web-based food frequency questionnaire explained elsewhere [25]. Iodine, ranging from 150 µg to 200 µg, was used in combination with other micronutrients, vitamins, with or without omega-3 fatty acids, by all users. Use of iodine supplement was coded yes or no employing compiled data from frequency questions (‘never’ was coded ‘no’, while ‘1–3 times per month’, ‘1–3 times per week’, ‘4–6 times per week’, and ‘daily’ were coded ‘yes’).

### 2.4. Outcome Variables—Neurocognitive Development

The cognitive, language, and motor scales of the Bayley Scales of Infant and Toddler Development, third edition (Bayley-III) were used to assess the infants’ developmental skills [28]. Bayley-III is an individually administered instrument designed to measure the developmental functioning of infants and toddlers, especially suited to detect developmental delays. The Bayley-III is commonly regarded as the gold standard in infant development assessment, provide good reliability and validity, and is often used as the instrument to validate other instruments [29,30]. At six and 12 months, the screening version of the test was used measuring all five domains: cognitive, receptive communication, expressive communication, gross motor and fine motor. At 18 months, the full-scale version was used measuring cognitive and language skills. The screening version is shorter than the full version comprising a selection of the test items employed in the full version. The two language scales are receptive communication, measuring verbal comprehension; and expressive communication, such as babbling, utterances, and gestures. Health care nurses, trained under supervision of clinical psychologists specialized in infant and child development, administered the test at the well-baby clinics. The parents were present in the room but instructed not to interfere with the testing. 

### 2.5. Covariates

When recruited in pregnancy, participants completed a questionnaire onsite at their local public health clinic and a questionnaire at home including questions on age, pre-pregnancy weight and height, parity, education, marital status, use of iodine containing supplements, use of omega-3 supplements in pregnancy, and daily smoking in pregnancy [25]. When infants were born, public health care nurses recorded data including child gender. 

### 2.6. Laboratory Analysis

Urine was collected at each site and stored at −18 °C pending analysis. Prior to analysis, the urine samples were defrosted in a refrigerator. UIC was determined by inductively coupled plasma mass-spectrometry (ICP-MS) after dilution with 1% tetrametylammonium hydroxid (TMAH). Prior to the analysis the samples were filtrated using a sterile membrane filter (0.45 µm pore size) and transferred to tubes appropriate for the analysis by the Agilent 7500 for ICP-MS at Institute of Marine Research, Bergen, Norway. Samples were analysed against a urine calibration curve (standard addition curve) to measure the unknown iodine concentration (127I) in the collected urine samples. Accuracy was verified with certified reference material; Seronorm Trace Elements Urine (Nycomed Pharma, Norway), iodine content: 84 µg/L (range, 72–96 µg/L) and 304 µg/L (range: 260–348 µg/L). In addition to UIC, I/Cr ratio and estimated 24 h UIE were determined. Determination of urinary creatinine concentration was analysed using the MAXMAT PL II multidisciplinary diagnostic platform with creatinine PAP kit [31].

### 2.7. Ethics

The trial complies with the Declaration of Helsinki and was commenced after approval by the Regional Committees for Medical and Health Research Ethics (2011/560 REK South-East). Written informed consent was obtained from the participants who could withdraw from the study at any time.

### 2.8. Statistical Analyses

Continuous variables are expressed as mean with SD for normally distributed data, as median with interquartile range (IQR) for non-normally distributed data, and categorical variables as numbers and percentages. ANOVA was used to compare numerical data and Pearson Ӽ2 to compare categorical data in Table 1 on background characteristics. 

Missing data on pre pregnancy body mass index (BMI) (*n* = 155 (18%)) were imputed by multiple imputation by chained equations in STATA, and 50 imputed datasets were generated for analyses. The imputation models included all variables included in the final analyses and was done separately for different exposures (UIC per liter, UIC per gram creatinine, UIC adjusted for creatinine by the residual method, and iodine supplement use in pregnancy). More details about the imputation models are provided in Method S1.

Multivariable mixed models were used to explore associations between exposures and repeated measures of cognitive score, receptive language skills, expressive language skills, fine motor skills, and gross motor skills. Associations between UIC and outcomes were modelled flexibly by use of restricted cubic splines (three knot positions, at percentiles 10, 50, and 90 of UIC). Potential time-varying effects were explored by testing interaction terms of exposure and time equal to zero. Outcome measures were standardized at each time point to obtain similar scales for the measurements. Since the mean and the variance at each time point by design were equal, generalized estimating equations were used to estimate the marginal effects, and an unstructured correlation matrix was specified to account for correlations between the repeated measurements. Results were reported as estimated mean across measurement occasions including 95% robust confidence intervals (CI). P-values for the overall associations of UIC and outcomes were calculated by testing all spline-coefficients equal to zero, and non-linearity by testing the second spline coefficient equal to zero.

We adjusted for potential confounding factors including maternal age, pre -pregnancy BMI, maternal education, marital status, parity, smoking in pregnancy, and included child gender, an important determinant of the outcomes, to increase the precision of the estimates. The sensitivity analysis on iodine containing supplement use also included use of omega-3 supplements as a covariate.

Two-tailed *p* < 0.05 was considered statistically significant. Statistical analyses of background characteristics were performed using Statistical Package for the Social Sciences (SPSS^®^ Statistics Version 24). All remaining analyses were performed in STATA (version 15.0; Stata Corp., College Station, TX, USA). The package postrcspline for STATA was used for graphing of the flexible models [32].

## 3. Results

### 3.1. Background Characteristics

Mean gestational week for collection of urine sample used in the analyses was 23.7 ± 4.9. The median UIC in pregnancy was 78 µg/L (IQR 46–130, min 4, max 750). In total, 676 (79%) women had a UIC of less than 150 µg/L and 242 (28%) women had a UIC of less than 50 µg/L (Table 1). The ages (mean ± SD, (min-max)) in infancy and toddlerhood at the three time points for neurodevelopmental assessment were 6.1 ± 0.4 (4.4–8.2), 12.2 ± 0.6 (10.1–15.6), and 18.4 ± 0.8 (16.7–22.2) months, respectively. There was minimal loss to follow up (mean number of assessments were 2.7 of 3 for cognitive scores and language scores, and 1.9 of 2 for fine and gross motor scores). 

In total, 155 (18%) women reported use of an iodine-containing supplement during the last three months before the spot-UIC was collected in pregnancy. Of the supplement users, daily use was reported by 69%, 4–6 days per week by 13.5%, 1–3 times per week by 12.3%, and, 1–3 times per month by 5.2%. The median (IQR) dose of iodine supplementation was 175 (25) µg. Considering the individual intake frequency this gives a median estimated daily intake of 150 (50) µg iodine from supplements. UIC was higher in supplement users (median 92 µg/L) compared to non-supplement users (median 77 µg/L), *p* < 0.001) (Table 1). 

### 3.2. Urinary Iodine Concentration and Neurodevelopment

There was a positive correlation between UIC (µg/L) and UIC/creatinine (µg/g), rs = 0.54, *p* < 0.001, a strong positive correlation between UIC (µg/L) and UIC~creatinine (µg/L), rs = 0.73, *p* < 0.001, and even stronger positive correlation between UIC~creatinine (µg/L) and UIC/creatinine (µg/g), rs = 0.93, *p* < 0.001.

**Figure 2:**
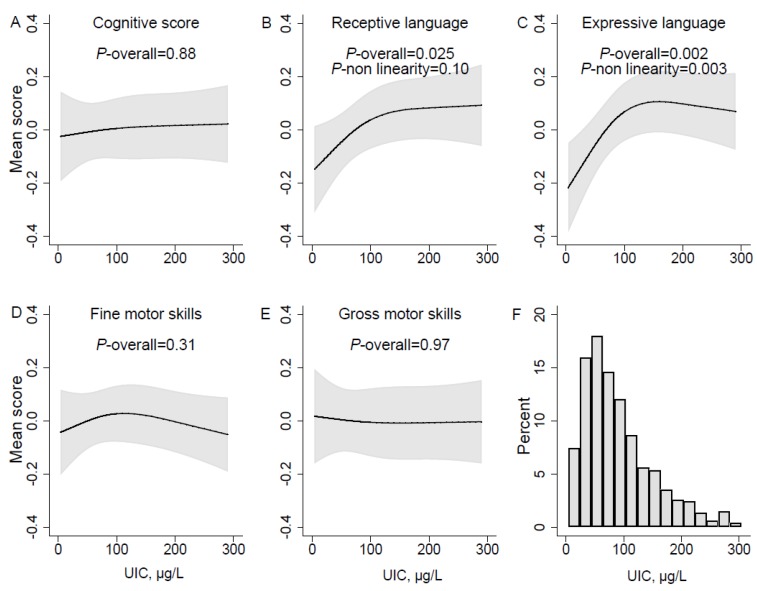
UIC µg/L in pregnancy and mean neurodevelopmental scores estimated in mixed models over sampling occasions at child age 6, 12, and 18 months. Panel A-E represent different neurodevelopmental domains, (**A**) Cognitive score; (**B**) Receptive language; (**C**) Expressive language; (**D**) Fine motor skills; (**E**) Gross motor skills. The histogram in panel (**F**) illustrates the distribution of urinary iodine concentration (µg/L) in the population. Associations were modelled flexibly by use of restricted cubic splines (three knot positions, at percentiles 10, 50, and 90 of UIC). Solid lines represent estimated means across measurement occasions, and the grey areas illustrate 95% robust confidence intervals (CI). P-values for the overall associations of UIC and outcomes were calculated by testing all spline-coefficients equal to zero, and non-linearity by testing the second spline coefficient equal to zero. Models are adjusted for maternal age, prepregnancy BMI, marital status, education, parity, daily smoking in pregnancy, and child sex.

Having a low UIC (µg/L) in pregnancy (lower than ~100 µg/L) was significantly associated with poorer skills in language domains (receptive and expressive) in infancy and toddlerhood, but not with poorer cognitive score or fine- and gross motor skills (Figure 2A–E). The adjusted associations for both language outcomes displayed similar curve-shapes, but non-linearity was only significant for expressive language (*p* = 0.003) (Figure 2C).

The associations with both language outcomes were also significant in the unadjusted analysis (Figure S1). There was no evidence of interactions with time for any of the outcomes, meaning there was no evidence of differences in the effects at different ages in infancy and toddlerhood. The association curves looked similar for the alternative exposures of UIC adjusted for creatinine, but did not reach statistical significance (Figure S2 and Figure S3). All analyses were repeated including only complete cases, i.e. excluding *n* = 117 participants with missing information on pre-pregnancy BMI, and it did not change the results (Figure S4 and Table S1).

### 3.3. Iodine Supplement use and Neurodevelopment

Maternal use of iodine-containing supplements in pregnancy was associated with poorer gross motor skills, standardized beta = −0.18, 95% CI = −0.33, −0.03, *p* = 0.02 (Table 2).

## 4. Discussion

The main finding of this study is that mild to moderate ID during pregnancy may adversely affect neurodevelopment in infancy and toddlerhood. The study population was sufficiently large to conclude that the median UIC indicates insufficient iodine intake in this population of pregnant women in Norway [33]. This is to our knowledge the largest and most complete assessment to date of maternal iodine urinary status during pregnancy and its relation to repeated measures of infant and toddler neurodevelopment.

### 4.1. Urinary Iodine Concentration and Neurodevelopment

We found that an insufficient iodine intake in pregnancy, reflected in a UIC below ~100 µg/L, was associated with lower infant language skills up to 18 months. This accords with studies in older children and suggests that inadequate iodine intake in pregnancy has consequences already in early in life. In the ALSPAC study, Bath et al. [12] found that children of mothers with UIC < 150 µg/g creatinine were more likely to score within the lowest quartile on verbal IQ, reading accuracy, and reading comprehension at age 8-9 years than children of mothers with UIC ≥ 150 µg/g creatinine. In a similar study in Australia, mild ID (i.e., UIC < 150 µg/L) in pregnancy was associated with lower educational outcomes in children at age 9 years [34]. A prospective study from The Netherlands reported that low maternal UIC during pregnancy (< 10th percentile) was associated with impaired executive function in children at age four years [35]. In MoBa, Abel et al. [36] concluded that maternal iodine intake below the Estimated Average Requirement during pregnancy (160 µg/day) was associated with increased risk of child language delay, more behavior problems, and reduced fine motor skills at age 3. In a recent publication from The UK Robinson et al. found a positive association between iodine status before conception (I/Cr 108 µg/g) and child IQ [14]. The results from our study support these findings and expand current knowledge by indicating that adverse effects on inadequate iodine intake on neurodevelopment is detectable already in infancy.

Why we see an effect on language skills but not cognition is unclear, and further data are needed. A substantial body of research points to many interrelations between language development and cognition [37]. It may be speculated that poor language skills are more easily detected at this early age than cognitive impairment, or that language and cognition are undifferentiated at this age [30]. The fact that iodine status was found not to be significantly associated with cognition in this study could be due to lack of power in the study (too few participants and a high degree of measurement error on outcomes at such a young age). One can see that the cognition graph has the same shape as the language skill graphs, but the association was not significant. Nevertheless, our findings are in line with previous research, including Hynes et al. [38], who found that a group of adolescents had poorer performance and continued to lag behind their peers with respect to language and literacy development 15 years after being exposed to mild ID in pregnancy.

The associations we explored in this study were similar, but no longer statistically significant when UIC was adjusted for creatinine. Creatinine adjustment is commonly used to remove variation in UIC due to hydration status. However, including creatinine in the estimation of the exposure variable might also introduce bias since creatinine excretion is not constant between individuals, but vary with e.g., age, BMI, muscle mass/fitness level, and gestational age [6]. Consequently, creatinine-adjusted UIC is associated with maternal characteristics that are potential predictors of the outcomes.

### 4.2. Iodine Supplement Use and Neurodevelopment

We did not find evidence of a protective effect of iodine supplement use in pregnancy. On the contrary, maternal iodine supplement use was associated with lower infant gross motor skills. This finding may be due to chance since we did not control for multiple comparisons. Since only 18% of the participants reported use of iodine-containing supplements, there was limited power to detect differences between the groups. We assessed iodine supplement use over the past three months before the first UIC and it covered primarily the second trimester. Ideally, we should have had data from before pregnancy and in the first trimester. Consequently, it cannot be ruled out that iodine supplement use was initiated too late to counteract the detrimental impact of inadequate maternal iodine status in the most critical time of fetal brain development. Results from studies of iodine supplement use in pregnancy in areas of mild- to moderate ID show conflicting results, and more research is needed [39]. Our findings of no benefit are supported by recent results from in MoBa [36,40] where it was found that the use of iodine supplements during pregnancy had no beneficial effect on the children’s neurodevelopment. Also, in a recently published RCT from India and Thailand, daily iodine supplementation in mildly iodine-deficient pregnant women (median UIC: 131 µg/L) had no effect on child neurodevelopment at age 5–6 years [41]. Altogether, this suggests a superior effect of habitual/long-term iodine intake from food, and that poor iodine intake and status before pregnancy cannot be compensated for by iodine-containing supplements in pregnancy.

### 4.3. Strengths and Limitations

A major strength of this study is the relatively large study population with data on UIC, the repeated measures of individually administered assessment of infant neurodevelopmental outcomes, and the minimal loss to follow up. An important limitation to the study is the use of a single spot-UIC which is not regarded as a good measure of iodine status at an individual level and thus it limits the statistical power when exploring associations to outcomes [6,42,43,44]. Observational studies tend to have a larger proportion of participants from higher socioeconomic status, introducing selection bias. However, associations between exposures and outcomes are not necessarily biased [45]. In this study population, however, there was a considerable proportion also of low-income mothers. Interestingly, we did not find any associations between UIC and education level or income. However, the observational design means we cannot rule out the possibility of residual confounding.

### 4.4. Implications of Findings

This study provides supporting evidence that mild to moderate ID in pregnancy impairs brain development and should be prevented. Reduced communication skills can influence the child’s possibilities to obtain information about the environment and is an early predictor of later school performance and IQ [46]. Consequently, poorer communication skills may reduce the capacity for the child to reach its developmental potential. Insufficient iodine status in pregnancy is a public health concern and actions are urgently needed. It could be questioned whether the cut-off for median UIC defining adequate iodine intake in pregnancy by the WHO of 150 µg/L (corresponding to an intake of 250 µg/day) is too high [1]. Our results, and also results from MoBa [36,40], suggests that a cutoff of UIC ~100 µg/L (or intake of ~160 µg/day) would be adequate. Recommendations for pregnant women should not be higher than necessary since securing an adequate iodine intake for pregnant women by food fortification may put other population groups at risk of iodine excess [47].

## 5. Conclusions

This study provides further evidence for a potential negative impact of insufficient iodine status in pregnancy on child neurodevelopment. The results showed that the tipping point for adverse developmental outcomes was UIC ~100 µg/L. Furthermore, we found no indication of a protective effect of iodine-containing supplements used in pregnancy, however, RCTs are needed to elucidate the impact of iodine supplement use. Preventing mild- to moderate ID in pregnant women by securing adequate iodine status before conception is an optimal strategy, since use of iodine containing supplements in pregnancy may be too late to counteract the detrimental effects of inadequate iodine intake.

## Figures and Tables

**Table 1:** Characteristics of the study population by UIC in pregnancy and by use of iodine-containing supplements.

Characteristic		UIC in Pregnancy (µg/L)					Iodine Supplement		
	*n*	All	<50	50–149	≥150	*p*-Value ^1^	Non Users	Users	*p*-Value ^2^
*n*	851	851	242	434	175		658	155	
UIC, µl/L ^3,4^,	851	78 (46,130)	33 (24,42)	83 (66,110)	200 (160,270)		77 (50,120)	92 (56,200)	<0.001
UIC, µg/g creatinine ^3,4^	851	82 (53,124)	57 (38,86)	77 (53,110)	143 (105,222)		75 (44,120)	98 (59,190)	<0.001
UIC~Cr, µg/l ^3,4^	851	81 (58,120)	63 (45,77)	80 (58,102)	178 (147,238)		78 (57,109)	110 (74,178)	<0.001
Age of mother (ys), mean ± SD	851	30.3 ± 4.7	30.2 ± 7.4	30.2 ± 4.5	30.7 ± 5.1	0.39	30.3 ± 4.6	30.2 ± 4.9	0.71
BMI, mean ± SD	696	23.4 ± 4.0	23.1 ± 3.7	23.6 ± 4.1	23.5 ± 4.3	0.40	23.4 ± 3.8	23.6 ± 4.8	0.60
Parity, %	851					0.15			0.38
Nulliparous	471	55	60	54	53		55	61	
Primiparous	275	32	26	35	34		33	28	
Multiparous	105	12	14	11	13		12	10	
Maternal education, %	851					0.39			0.37
Low (≤ 12 years)	182	21	24	22	16		21	17	
Medium (13–16 years)	323	38	38	37	41		38	43	
High (≥ 17 years)	346	41	39	41	43		41	41	
Maternal cohabitation, %	851					0.71			0.23
Yes	829	97	97	97	98		97	99	
No	22	2.6	2.9	2.8	1.7		3.0	1.3	
Iodine containing supplement, %	813								
Yes	155	19	12	18	31	<0.001	Na	Na	Na
No	658	81	88	82	69		Na	Na	
Daily smoking in pregnancy, %	851					0.30			0.76
Yes	24	2.8	4.1	2.1	2.9		97	97	
No	827	97	96	98	97		3.0	2.6	
Infant gender, %	851					0.60			0.69
Boys	433	51	50	52	49		52	50	
Girls	418	49	50	48	51		48	50	

^1^ Comparison of numerical data with ANOVA and categories with pearson Ӽ2.^2^ Comparison of numerical data with student’s t-test (UIC-measures were log transformed) and categories with pearson Ӽ2. ^3^ Results reported as median (IQR) for all participants and UIC categories. ^4^ Values are log transformed and reported as mean ± SD. Abbreviations: UIC, Urinary iodine concentration; BMI, body mass index; Na, not applicable.

**Table 2:** Longitudinal regressions of maternal use of iodine-containing supplement and the overall mean of standardized scores on child neurodevelopment. Non-users of iodine-containing supplements serve as reference group.

		Iodine Supplement					
Bayley III ^1^	*n*	Crude Models			Adjusted Models ^2^		
		B ^3^	95% CI	*p*	B ^3^	95% CI	*p*
Cognitive score	813	0.00	−0.13, 0.13	0.98	0.02	−0.11, 0.15	0.79
Receptive language	813	0.02	−0.10, 0.14	0.79	−0.02	−0.14, 0.11	0.81
Expressive language	813	0.07	−0.05, 0.20	0.26	0.05	−0.08, 0.18	0.44
Fine motor skills	810	−0.12	−0.25, 0.02	0.093	−0.10	−0.23, 0.03	0.15
Gross motor skills	810	−0.20	−0.35, −0.05	0.010	−0.18	−0.33, −0.03	0.020

^1^ Bayley Scales of Infant and Toddler Development, third edition. ^2^ Models were adjusted for maternal age, prepregnancy BMI, marital status, education, parity, daily smoking in pregnancy, use of supplements containing omega-3 fatty acid in pregnancy, and child sex. ^3^ Standardized beta coefficient. B represents the standardized beta, i.e. the estimated difference in mean outcome score (in standard deviations) between the exposed and the non-exposed (to iodine supplement use).

## Supplementary Materials

The following are available online at http://www.mdpi.com/2072-6643/10/9/1270/s1, Method S1: Imputation; Table S1: Longitudinal regressions of maternal use of iodine-containing supplement and the overall mean of standardized scores on child neurodevelopment. Non-users of iodine-containing supplements serve as reference group. Complete case analysis; Figure S1: UIC µg/L in pregnancy and the overall mean of standardized neurodevelopmental scores in infancy and toddlerhood. Panel A-E represent different neurodevelopmental domains, A: Cognitive score; B: Receptive language; C: Expressive language; D: Fine motor skills; E Gross motor skills. The histogram in panel F illustrates the distribution of urinary iodine concentration (µg/L) in the population. Associations were modelled flexibly by use of restricted cubic splines (three knot positions, at percentiles 10, 50, and 90 of UIC). Solid lines represent estimated means across measurement occasions, and the grey areas illustrate 95% robust confidence intervals (CI). P-values for the overall associations of UIC and outcomes were calculated by testing all spline-coefficients equal to zero, and non-linearity by testing the second spline coefficient equal to zero. Models are crude; Figure S2: UIC~Cr, µg/l in pregnancy and the overall mean of standardized neurodevelopmental scores in infancy and toddlerhood. Panel A-E represent different neurodevelopmental domains, A: Cognitive score; B: Receptive language; C: Expressive language; D: Fine motor skills; E Gross motor skills. The histogram in panel F illustrates the distribution of urinary iodine concentration (µg/L) in the population. Associations were modelled flexibly by use of restricted cubic splines (three knot positions, at percentiles 10, 50, and 90 of UIC). Solid lines represent estimated means across measurement occasions, and the grey areas illustrate 95% robust confidence intervals (CI). P-values for the overall associations of UIC and outcomes were calculated by testing all spline-coefficients equal to zero, and non-linearity by testing the second spline coefficient equal to zero. Models are crude; Figure S3: UIC, µg/g creatinine and the overall mean of standardized neurodevelopmental scores in infancy and toddlerhood. Panel A-E represent different neurodevelopmental domains, A: Cognitive score; B: Receptive language; C: Expressive language; D: Fine motor skills; E Gross motor skills. The histogram in panel F illustrates the distribution of UIC (µg/g) in the population. Associations were modelled flexibly by use of restricted cubic splines (three knot positions, at percentiles 10, 50, and 90 of UIC). Solid lines represent estimated means across measurement occasions, and the grey areas illustrate 95% robust confidence intervals (CI). P-values for the overall associations of UIC and outcomes were calculated by testing all spline-coefficients equal to zero, and non-linearity by testing the second spline coefficient equal to zero. Models are adjusted for maternal age, prepregnancy BMI, marital status, education, parity, daily smoking in pregnancy, and child sex. The solid line is the dose–response curve; the grey area represents the 95% confidence interval; Figure S4: UIC µg/L in pregnancy and mean neurodevelopmental scores estimated in mixed models over sampling occasions at child age 6, 12, and 18 months. Panel A-E represent different neurodevelopmental domains, **A**: Cognitive score; **B**: Receptive language; **C**: Expressive language; **D**: Fine motor skills; **E:** Gross motor skills. The histogram in panel **F** illustrates the distribution of urinary iodine concentration (µg/L) in the population. Associations were modelled flexibly by use of restricted cubic splines (three knot positions, at percentiles 10, 50, and 90 of UIC). Solid lines represent estimated means across measurement occasions, and the grey areas illustrate 95% robust confidence intervals (CI). P-values for the overall associations of UIC and outcomes were calculated by testing all spline-coefficients equal to zero, and non-linearity by testing the second spline coefficient equal to zero. Models are adjusted for maternal age, prepregnancy BMI, marital status, education, parity, daily smoking in pregnancy, and child sex. Complete case analysis.
