# Supplementary material for: Maternal Iodine Status is Associated with Offspring Language Skills in Infancy and Toddlerhood
Source: Nutrients. 2018 Sep 9;10(9):1270. doi: 10.3390/nu10091270 (PMC6163597; doi:10.3390/nu10091270)
Supplement: Supplementary file 1 [file nutrients-10-01270-s001.zip › Figure Captions for Supplementary Figures.pdf]

Figure Captions for Supplementary Figures

Figure S1: UIC µg/L in pregnancy and the overall mean of standardized neurodevelopmental scores in infancy and toddlerhood. Panel A-E represent different neurodevelopmental domains, A: Cognitive score; B: Receptive language; C: Expressive language; D: Fine motor skills; E Gross motor skills. The histogram in panel F illustrates the distribution of urinary iodine concentration (µg/L) in the population. Associations were modelled flexibly by use of restricted cubic splines (three knot positions, at percentiles 10, 50, and 90 of UIC). Solid lines represent estimated means across measurement occasions, and the grey areas illustrate 95% robust confidence intervals (CI). P-values for the overall associations of UIC and outcomes were calculated by testing all spline-coefficients equal to zero, and non-linearity by testing the second spline coefficient equal to zero. Models are crude.

Figure S2: UIC~Cr, µg/l in pregnancy and the overall mean of standardized neurodevelopmental scores in infancy and toddlerhood. Panel A-E represent different neurodevelopmental domains, A: Cognitive score; B: Receptive language; C: Expressive language; D: Fine motor skills; E Gross motor skills. The histogram in panel F illustrates the distribution of urinary iodine concentration (µg/L) in the population. Associations were modelled flexibly by use of restricted cubic splines (three knot positions, at percentiles 10, 50, and 90 of UIC). Solid lines represent estimated means across measurement occasions, and the grey areas illustrate 95% robust confidence intervals (CI). P-values for the overall associations of UIC and outcomes were calculated by testing all spline-coefficients equal to zero, and non-linearity by testing the second spline coefficient equal to zero. Models are crude.

Figure S3: UIC, µg/g creatinine and the overall mean of standardized neurodevelopmental scores in infancy and toddlerhood. Panel A-E represent different neurodevelopmental domains, A: Cognitive score; B: Receptive language; C: Expressive language; D: Fine motor skills; E Gross motor skills. The histogram in panel F illustrates the distribution of UIC (µg/g) in the population. Associations were modelled flexibly by use of restricted cubic splines (three knot positions, at percentiles 10, 50, and 90 of UIC). Solid lines represent estimated means across measurement occasions, and the grey areas illustrate 95% robust confidence intervals (CI). P-values for the overall associations of UIC and outcomes were calculated by testing all spline-coefficients equal to zero, and non-linearity by testing the second spline coefficient equal to zero. Models are adjusted for maternal age, prepregnancy BMI, marital status, education, parity, daily smoking in pregnancy, and child sex. The solid line is the dose–response curve; the grey area represents the 95% confidence interval.

Figure S4: UIC µg/L in pregnancy and mean neurodevelopmental scores estimated in mixed models over sampling occasions at child age 6, 12, and 18 months. Panel A-E represent different neurodevelopmental domains, A: Cognitive score; B: Receptive language; C: Expressive language; D: Fine motor skills; E: Gross motor skills. The histogram in panel F illustrates the distribution of urinary iodine concentration (µg/L) in the population. Associations were modelled flexibly by use of restricted cubic splines (three knot positions, at percentiles 10, 50, and 90 of UIC). Solid lines represent estimated means across measurement occasions, and the grey areas illustrate 95% robust confidence intervals (CI). P-values for the overall associations of UIC and outcomes were calculated by testing all spline-coefficients equal to zero, and non-linearity by testing the second spline coefficient equal to zero. Models are adjusted for maternal age, prepregnancy BMI, marital status, education, parity, daily smoking in pregnancy, and child sex. Complete case analysis.
