# Supplementary material for: Maternal Iodine Status is Associated with Offspring Language Skills in Infancy and Toddlerhood
Source: Nutrients. 2018 Sep 9;10(9):1270. doi: 10.3390/nu10091270 (PMC6163597; doi:10.3390/nu10091270)
Supplement: Supplementary file 1 [file nutrients-10-01270-s001.zip › Method S1.pdf]

Method S1: Imputation

Pre-pregnancy BMI was imputed using the predictive mean matching imputation method (pmm). Some participants had missing on one or more of the 13 outcome measurements (35%) or had not participated in one or more assessments, and these missing values were first imputed in the MICE-model (by pmm for outcome variables and by the Gaussian normal regression imputation method (regress) for age at assessment), and then recoded to missing. The method we have chosen for imputing the missing BMI values is built on the assumption that the missing values are missing at random (i.e. the missingness is not dependent on the true value of BMI after controlling for the other variables in the models that are not missing). However, we cannot exclude the possibility that some women had not reported their weight and height in the questionnaire because their BMI were was not optimal. Results from adjusted models based on complete cases (i.e. with no missing covariates) are presented in Figure S1 and Table S1. The results were not changed when only complete cases were included (n=696 (694 for motor outcomes)). Also, if BMI was excluded as a covariate from all the models, the results remained unchanged. Thus, BMI was not an important confounder in these analyses.;
