# Supplementary material for: Maternal Iodine Status is Associated with Offspring Language Skills in Infancy and Toddlerhood
Source: Nutrients. 2018 Sep 9;10(9):1270. doi: 10.3390/nu10091270 (PMC6163597; doi:10.3390/nu10091270)
Supplement: Supplementary file 1 [file nutrients-10-01270-s001.zip › Table S1.pdf]

Table S1. Longitudinal regressions of maternal use of iodine-containing supplement and the overall mean of standardized scores on child neurodevelopment. Non-users of iodine-containing supplements serve as reference group. Complete case analysis.

		Iodine supplement								
Bayley III1	n	Crude models			Adjusted models[2]			Adjusted models[2] Complete cases*		
		B3	95% CI	p	B3	95% CI	p	B3	95% CI	p
Cognitive score	813	0.00	-0.13,0.13	0.98	0.02	-0.11,0.15	0.79	0.08	-0.05,0.22	0.24
Receptive language	813	0.02	-0.10,0.14	0.79	-0.02	-0.14,0.11	0.81	0.03	-0.10,0.16	0.67
Expressive language	813	0.07	-0.05,0.20	0.26	0.05	-0.08,0.18	0.44	0.10	-0.03,0.23	0.14
Fine motor skills	810	-0.12	-0.25,0.02	0.093	-0.10	-0.23,0.03	0.15	-0.06	-0.20,0.08	0.42
Gross motor skills	810	-0.20	-0.35,-0.05	0.010	-0.18	-0.33,-0.03	0.020	-0.17	-0.33,-0.01	0.033

[1]Bayley Scales of Infant and Toddler Development, third edition.

[2]Models were adjusted for maternal age, prepregnancy BMI, marital status, education, parity, daily smoking in pregnancy, use of supplements containing omega-3 fatty acid in pregnancy, and child sex.

[3]Standardized beta coefficient

* Models include only participants with no missing covariates (n=696 (694 for motor outcomes)).
