# Supplementary figures and images for: Maternal Iodine Status is Associated with Offspring Language Skills in Infancy and Toddlerhood
Source: Nutrients. 2018 Sep 9;10(9):1270. doi: 10.3390/nu10091270 (PMC6163597; doi:10.3390/nu10091270)

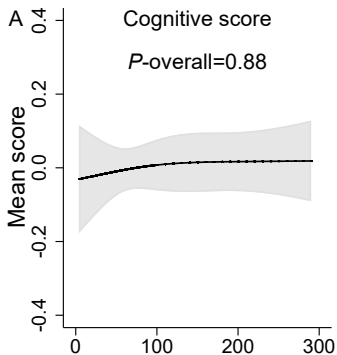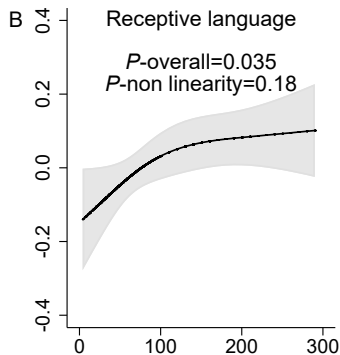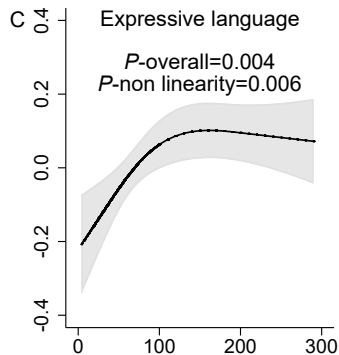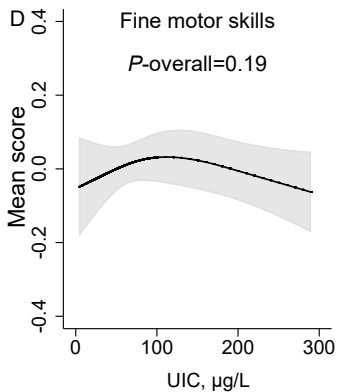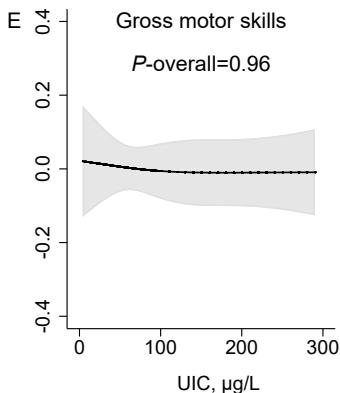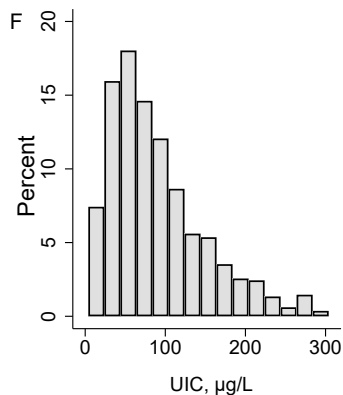

Supplement: Supplementary file 1 [file nutrients-10-01270-s001.zip › Figure S1.pdf]

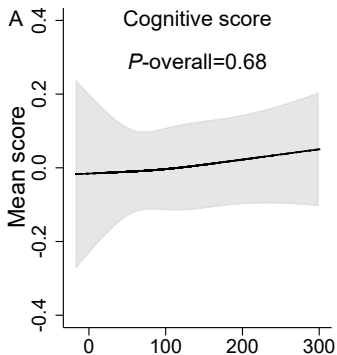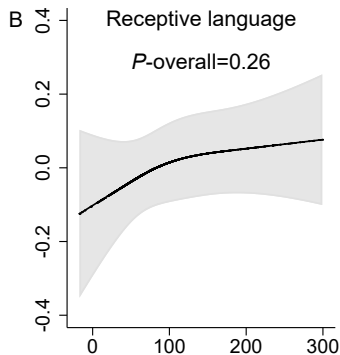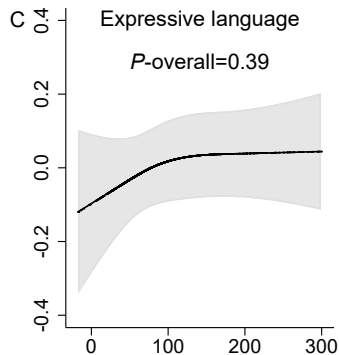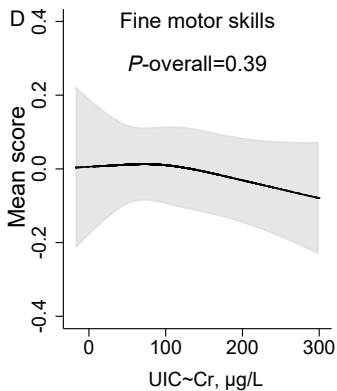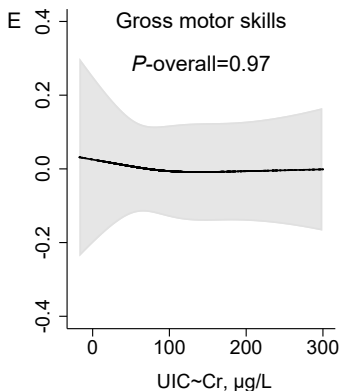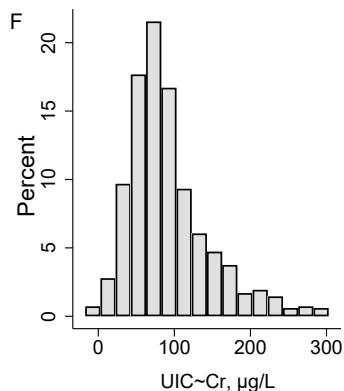

Supplement: Supplementary file 1 [file nutrients-10-01270-s001.zip › Figure S2.pdf]

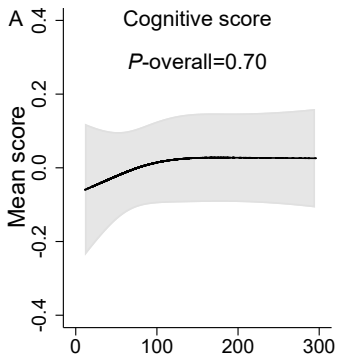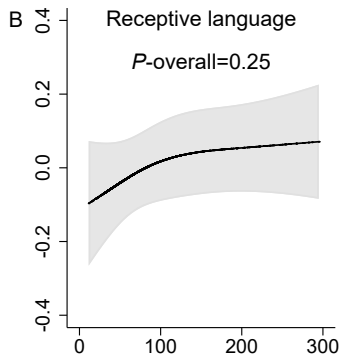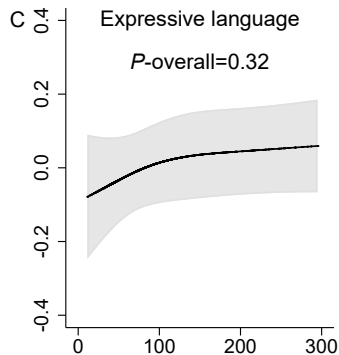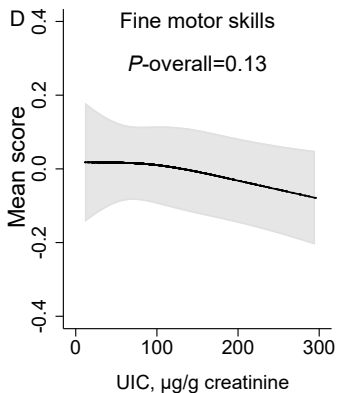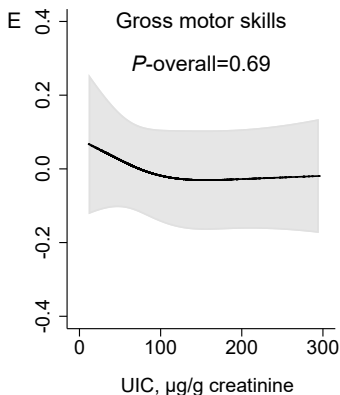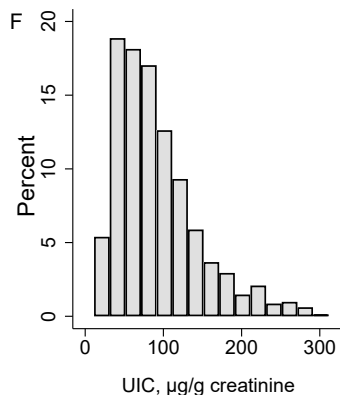

Supplement: Supplementary file 1 [file nutrients-10-01270-s001.zip › Figure S3.pdf]

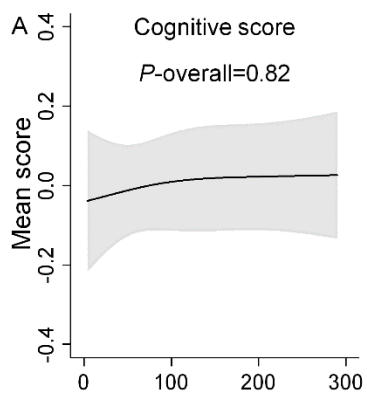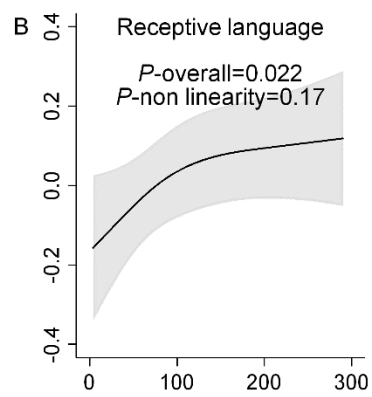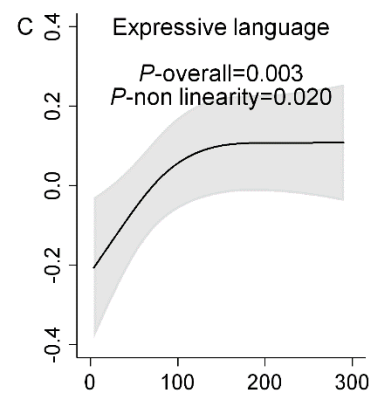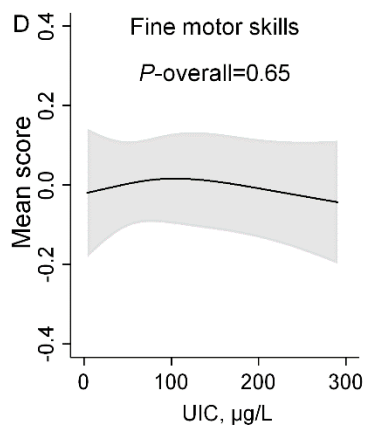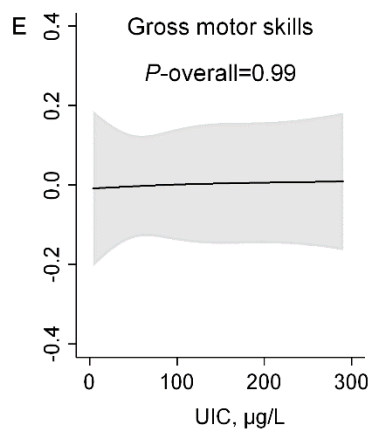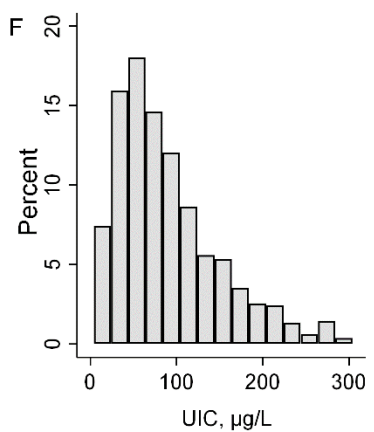

Supplement: Supplementary file 1 [file nutrients-10-01270-s001.zip › Figure S4.pdf]
